# Midlife Residential Segregation and Healthcare Access as Mediators of Education and Cognitive Impairment

**DOI:** 10.1093/geroni/igaf122.558

**Published:** 2025-12-31

**Authors:** Min Hee Kim

**Affiliations:** Rutgers, New Brunswick, New Jersey, United States

## Abstract

Despite increasing evidence documenting the causal effect of education in reducing the risk of Alzheimer’s disease and related dementias, few studies have quantified the cumulative (dis)advantage across the life course in action. This study applies a causal mediation analysis to unpack the causal effect of education on the risk of cognitive impairment risk via upstream and downstream pathways. We leverage changes in historical measures of state-level education policies as natural experiments and estimated the effects of educational attainment on cognitive impairment in the REasons for Geographic and Racial Disparities in Stroke (REGARDS) study, a national longitudinal survey of Black and White older adults aged 45+ with up to 20 years of follow-ups (N = 21,603). We decompose total effect into direct and indirect effects by mid-life exposures as mediators. Of particular interest are residential segregation and public health infrastructure and individual-level socioeconomic, stress, and healthcare access and quality conditions. Our sample includes 80% of respondents with diagnosed cardiometabolic conditions at baseline and 13% of respondents with experienced cognitive impairment incidence in the follow-up. We will discuss methodological challenges and advances in causal mediation (i.e., addressing multiple mediators and intermediate confounding). This study, which attempts to decompose some of the “synergistic impact” of policy and institutional choices, will facilitate further discussions on policy intervention points for reducing health disparities.

